# Multi-omics analysis reveals regulators of the response to PDGF-BB treatment in pulmonary artery smooth muscle cells

**DOI:** 10.1186/s12864-016-3122-3

**Published:** 2016-10-06

**Authors:** Jidong Chen, Xiaolei Cui, Zhengjiang Qian, Yanjiao Li, Kang Kang, Junle Qu, Li Li, Deming Gou

**Affiliations:** 1 Shenzhen Key Laboratory of Microbial Genetic Engineering, Shenzhen Key Laboratory of Marine Bioresource and Eco-environmental Science, College of Life Sciences and Oceanography, Shenzhen University, Nanhai Ave 3688, Shenzhen, Guangdong 518060 China; 2 Key Laboratory of Optoelectronic Devices and Systems of Ministry of Education and Guangdong Province, College of Optoelectronic Engineering Shenzhen University, Shenzhen, Guangdong 518060 China; 3 Department of Physiology, Shenzhen University Health Science Center, Shenzhen, Guangdong 518060 China

**Keywords:** Platelet-derived growth factor-BB (PDGF-BB), Pulmonary arterial hypertension (PAH), RNA sequencing, iTRAQ, BMPR2, miR-376b

## Abstract

**Background:**

Pulmonary arterial hypertension (PAH) is a lethal disease with pronounced narrowing of pulmonary vessels due to abnormal cell proliferation. The platelet-derived growth factor BB (PDGF-BB) is well known as a potent mitogen for smooth muscle cell proliferation. To better understand how this growth factor regulates pulmonary arterial smooth muscle cells (PASMCs) proliferation, we sought to characterize the response to PDGF-BB stimulation at system-wide levels, including the transcriptome and proteome.

**Results:**

In this study, we identified 1611 mRNAs (transcriptome), 207 proteins (proteome) differentially expressed in response to PDGF-BB stimulation in PASMCs based on RNA-sequencing and isobaric tags for relative and absolute quantification (iTRAQ) assay. Transcription factor (TF)-target network analysis revealed that PDGF-BB regulated gene expression potentially *via* TFs including HIF1A, JUN, EST1, ETS1, SMAD1, FOS, SP1, STAT1, LEF1 and CEBPB. Among them, SMAD1-involved BMPR2/SMADs axis plays a significant role in PAH development. Interestingly, we observed that the expression of BMPR2 was decreased in both mRNA and protein level in response to PDGF-BB. Further study revealed that BMPR2 is the direct target of miR-376b that is up-regulated upon PDGF-BB treatment. Finally, EdU incorporation assay showed that miR-376b promoted proliferation of PASMCs.

**Conclusion:**

This integrated analysis of PDGF-BB-regulated transcriptome and proteome was performed for the first time in normal PASMCs, which revealed a crosstalk between PDGF signaling and BMPR2/SMADs axis. Further study demonstrated that PDGF-BB-induced miR-376b upregulation mediated the downregulation of BMPR2, which led to expression change of its downstream targets and promoted proliferation of PASMCs.

**Electronic supplementary material:**

The online version of this article (doi:10.1186/s12864-016-3122-3) contains supplementary material, which is available to authorized users.

## Background

Pulmonary arterial hypertension (PAH) is a life-threatening disease characterized by a sustained elevation of pulmonary arterial pressure and pulmonary vascular resistance [1, 2]. Essential pathological characteristics of PAH are excessive proliferation and migration of pulmonary arterial smooth muscle cells (PASMCs), leading to medial hypertrophy and vascular remodeling [3, 4]. PASMCs are maintained in a quiescent and non-migratory state under normal condition. However, PASMCs proliferation and migration are significantly promoted in response to various growth factors and cytokines, such as platelet-derived growth factor-BB (PDGF-BB), fibroblast growth factor, insulin-like growth factor-1, tumor necrosis factor-α and interleukin-1 [5–7].

PDGF is the most potent mitogenic factor for vascular smooth muscle cells (VSMCs), and exerts its actions *via* binding and activating two PDGF receptor (PDGFR) subtypes, PDGFRα and PDGFRβ [8–10]. Abundant evidence reveals that PDGF is a major contributor to the pathobiology of vascular disorders including PAH [11, 12]. PDGF ligands are upregulated in lung tissue and pulmonary cells in monocrotaline (MCT)- and hypoxia-induced experimental PAH animal [13–16]. Similar alterations with upregulation of PDGF ligands and PDGFRβ were found in lung tissue and small pulmonary arteries of patients with PAH [17, 18]. Imatinib, a PDGF receptor antagonist, has been reported to dramatically improve PAH in some human cases as well as animal models, but serious side effects and drug discontinuation are common [19, 20]. Therefore, it is particularly necessary to investigate gene expression alteration globally in PASMCs induced by PDGF-BB.

Previously, Shirvani and his colleagues have globally determined the effect of PDGF on transcription factor CHF1/Hey2-knockout VSMCs and demonstrate that CHF1/Hey2 profoundly affects vascular smooth muscle phenotype by altering both the absolute expression level of a variety of genes and the kinetics of growth factor-induced gene expression. [21]. In another report, Lee et al. have analyzed the gene expression profile with multiple whole-genome expression array datasets and identified NFAT family members and target genes as important effectors of VSMCs in response to PDGF [22]. These studies were mainly based on traditional high throughput technology, such as microarray, which covered only portion of the whole genome [21, 22]. In addition, a recent study has integrated proteomic and transcriptomic profiles and identified a novel PDGF-MYC network in bladder smooth muscle cells [23], however, this kind of analysis in PASMCs is still lacking.

To better understand how PDGF-BB regulates gene expression in PASMCs, we performed the integrated analysis of transcriptome and proteome changes in response to PDGF-BB stimulation in rat PASMCs (RPASMCs), based on next generation sequencing and relative and absolute quantification (iTRAQ) technology. Our analysis revealed a significant role of SMAD1 in PDGF-BB induced gene expression. Further study demonstrated that PDGF-BB-induced miR-376b upregulation mediated the downregulation of BMPR2, which led to expression change of SMAD1 targets and promoted proliferation of PASMCs.

## Results

### Profiling of mRNA expression in response to PDGF-BB

**Fig. 1:**
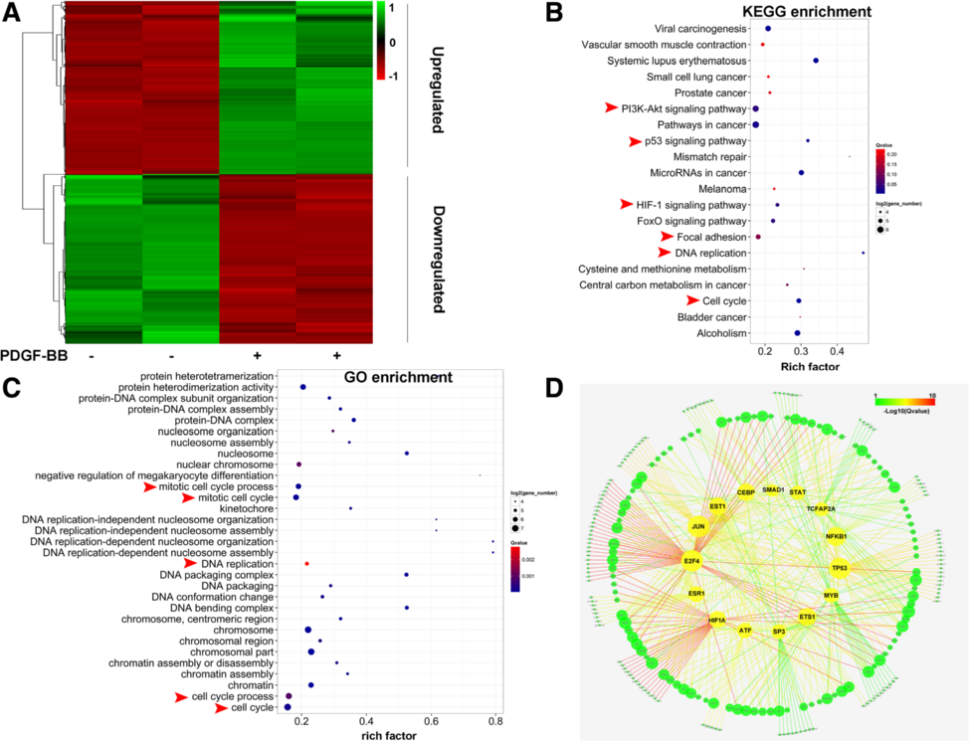
Transcriptome analysis of RPASMCs treated with PDGF-BB. **a**, RNA sequencing heatmap showing a subset of genes differentially expressed upon RPASMCs treated with PDGF-BB for 12 h (FC > 2, FKPM > 0.5 and Qvalue < 0.05 was shown); **b**, Gene ontology (GO) analysis was carried out on genes differentially expressed, heatmap showing total expression of genes in the most enriched GO terms; **c**, the most enriched pathways of differentially expressed genes, analyzing *via* KEGG pathway; **d**, The network was constructed with genes differentially expressed and their potential corresponded transcription factors (TFs), showing the 15 most enriched TFs (*yellow*), Color of line represented the enrichment level. As to a GO or KEGG term, Rich factor = (number of differentially expressed gene) / (total gene number), Qvalue is *p*-value adjusted by method “*Benjamini and Hochberg*”

RNA deep-sequencing was carried out to identify differentially expressed genes in response to PDGF-BB. Total RNA samples for RNA-Seq analysis were obtained from RPASMCs following PDGF-BB (30 ng/ml) exposure for 0 or 12 h (2 independently isolated biological replicates). Every sample generated more than 90,000,000 reads after quality control, and 50,553 mRNA transcripts were identified with 373 novel transcripts that have not been reported in rat. By quantitative analysis, 1611 transcripts were revealed to express differentially in response to PDGF-BB (Q value < 0.05; FPKM > 0.5; fold change (FC) ≥ 2), with 814 up- and 797 down-regulated (Fig. 1a and Additional file 1).

Next, gene ontology (GO) analysis was performed with KOBAS 2.0 online software (http://kobas.cbi.pku.edu.cn/) [24]. Results showed that these differentially expressed genes were enriched significantly in GO terms involved in cell proliferation, such as cell cycle, DNA replication and cell division (Fig. 1c and Additional file 2), suggesting the significant role of PDGF-BB in smooth muscle cell proliferation as reported previously [23]. KEGG (Kyoto Encyclopedia of Genes and Genomes) pathway analysis suggested that differentially expressed genes were enriched significantly in cell cycle, DNA replication, PI3K-Akt signaling pathway, HIF-1 signaling pathway, focal adhesion. All of these pathways are functionally involved in cell proliferation and migration (Fig. 1b and Additional file 2). To find out the potential transcription factors (TFs) mediating PDGF-BB regulation on these differentially expressed mRNA, TF-targets data were collected from TRED database [25] and compared to differentially expressed mRNAs. Data analysis revealed that E2F4, HIFA, JUN, TP53, ATF, ESR1, EST1, ETS1, SMAD1 and NFKB1 were top 10 TFs in response to PDGF-BB stimulation (Fig. 1d), partly consistent with previous reports on bladder smooth muscle cells [23]. Interestingly, we identified as the first time that SMAD1, an essential TF in BMPR2/SMADs signaling pathway was regulated by PDGF-BB (Fig. 1d), implying a potential link between BMPR2/SMADs signaling and PDGF-BB stimulation.

### Profiling of protein expression in response to PDGF-BB

**Fig. 2:**
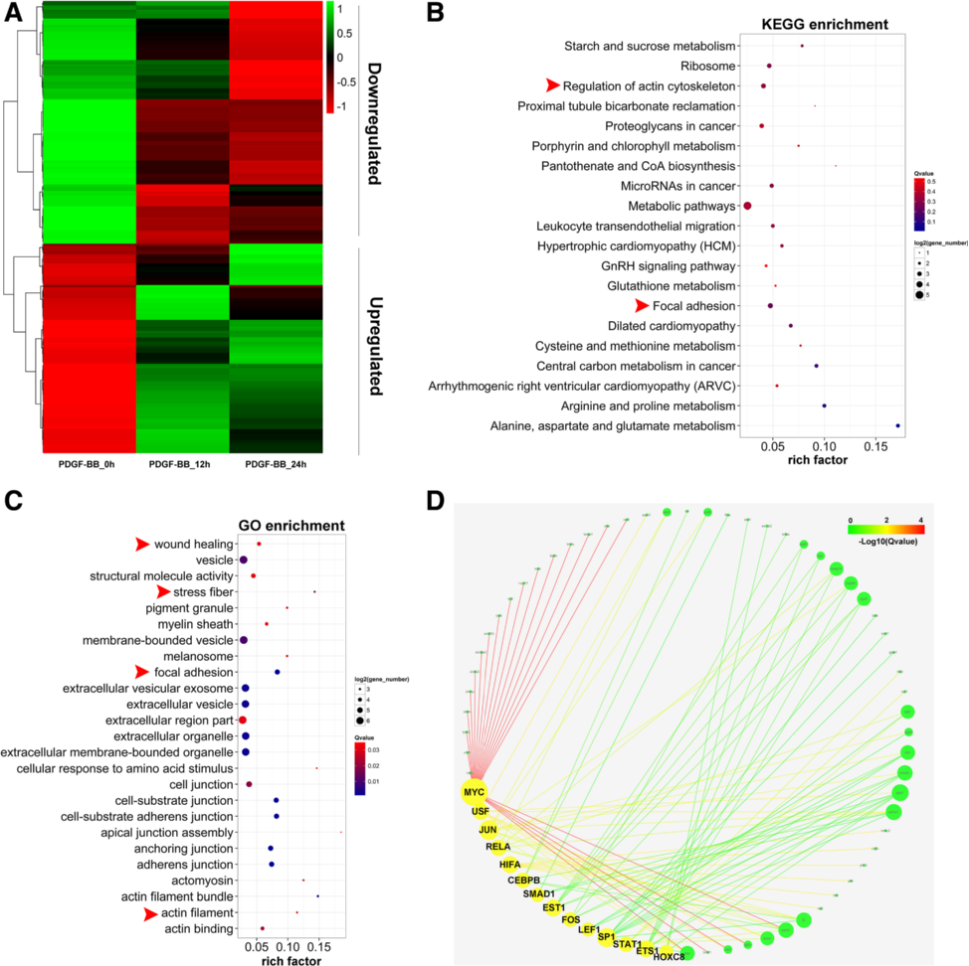
Protenome analysis of RPASMCs treated with PDGF-BB. **a**, heatmap displaying a subset of proteins differentially expressed upon RPASMCs treated 12 or 24 h with PDGF-BB (FC > 1.6, *p* <0.05), measured *via* iTRAQ; **b**, GO analysis showing the most enriched GO term on differentially expressed proteins in response to PDGF-BB; **c**, the most enriched pathway of proteins differentially expressed, analyzing *via* KEGG pathway; **d**, The network was constructed with differentially expressed proteins and their potential corresponded transcription factors (TFs), displaying the 14 significantly enriched TFs (*yellow*), Color of line represented the enrichment level. As to a GO or KEGG term, Rich factor = (number of differentially expressed gene) / (total gene number), Qvalue is *p*-value adjusted by method “*Benjamini and Hochberg*”

iTRAQ-based proteomics analysis was performed in RPASMCs treated with PDGF-BB in three time points (0, 12, 24 h, duplicate per time point). Totally, 3666 proteins were identified from 26,878 peptides matched with 82,825 spectra at a FDR (false discovery rate) of 5 %, 2934 of which matched to two or more peptides. Among them, 207 proteins were differentially expressed in response to PDGF-BB stimulation for 12 or 24 h (*p* < 0.05, FC > 1.6), with 96 up- and 111 down-regulated (Fig. 2a and Additional files 3 and 4).

GO analysis suggested the differentially expressed protein were also enriched in GO terms involved in proliferation and migration, such as focal adhesion, stress fiber and wound healing (Fig. 2b). In KEGG pathway analysis, only one pathway was showed to be enriched significantly. However, among the 10 most enriched pathways, focal adhesion and regulation of actin cytoskeleton were involved tightly in migration and proliferation (Fig. 2c). TF-target analysis suggested that differentially expressed proteins majorly enriched in the TFs including MYC, HIFA, USF, JUN and REL (Fig. 2d). Interestingly, differentially expressed proteins were enriched significantly in SMAD1 as in mRNA level (Fig. 2d).

### Comparison and integration of transcriptome and proteome

**Fig. 3:**
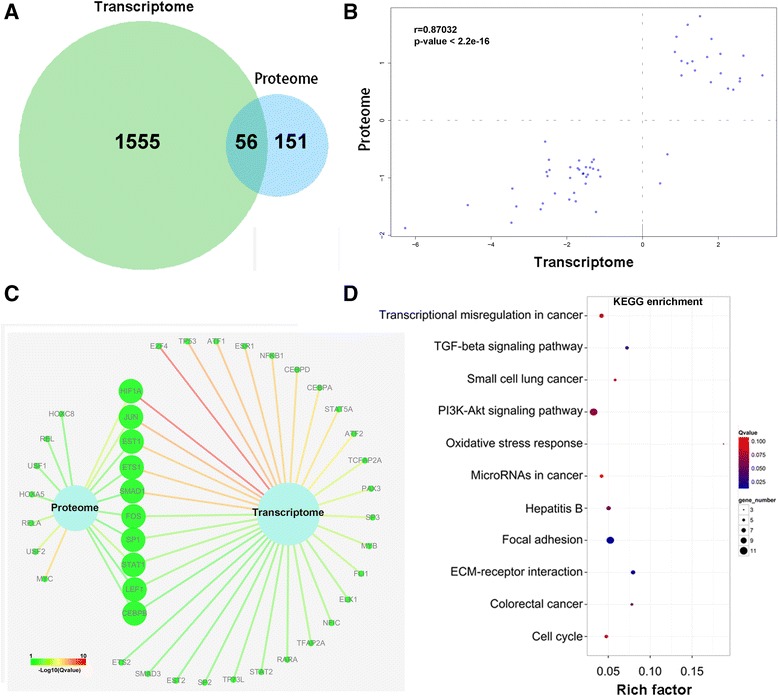
Comparison and integration of differential transcriptome and proteome. **a**, Venn diagram revealing 56 genes expressed differentially in both mRNA and protein levels; **b**, Differential expression of these 56 genes was significantly correlated (*r* = 0.87032, *p* < 0.001, with Pearson test); **c**, Integration of TF-target network at proteome and transcriptome levels, Color of line represented the enrichment level; **d**, the most enriched pathway of differentially expressed genes corresponding to SMAD family, analyzing *via* KEGG pathway. As to a KEGG term, Rich factor = (number of differentially expressed gene) / (total gene number), Q value is *p*-value adjusted by method “*Benjamini and Hochberg*”

To further identify PDGF-BB mediated gene expression, the datasets from RNA-seq and iTRAQ were applied for the integrated analysis. Totally, 56 genes were found to be differentially expressed at both mRNA and protein levels (Fig. 3a). The correlation analysis suggested that these genes expressed in high correlation in the two levels (*r* = 0.87032, *p* < 0.001, Fig. 3b). In addition, The integrated analysis of TFs-target suggested that differentially expressed genes enriched significantly in HIF1A, JUN, EST1, ETS1, SMAD1, FOS, SP1, STAT1, LEF1 and CEBPB, at both mRNA and proteins levels (Fig. 3c). It is well known that BMPR2/SMAD1 axis plays critical role during the PAH initiation and development [26, 27]. Hence we chose potential targets of SMADs for further analysis. KEGG pathway analysis suggested that these differentially expressed targets enriched significantly in PI3K-Akt signaling pathway, focal adhesion and cell cycle, and all these pathways play significant roles in cell proliferation and migration (Fig. 3d).

### Validation of differentially expressed mRNA identified *via* RNA-sequencing

**Fig. 4:**
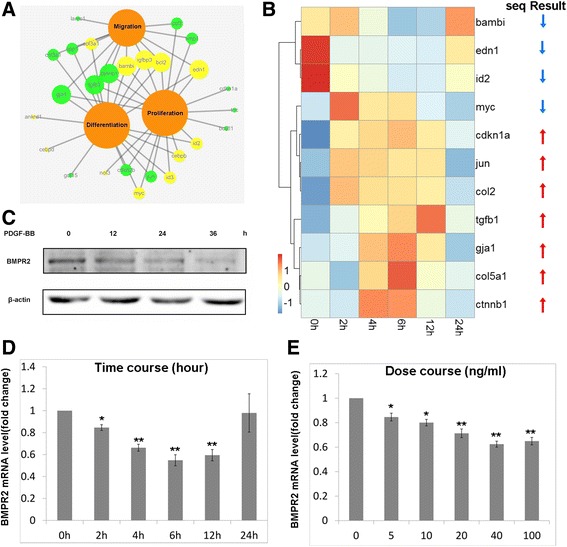
Differential expression of SMAD family TFs in response to PDGF-BB was due to downregulation of BMPR2. **a**, differentially expressed genes corresponding to SMAD involved in 3 major cellular procedures; **b**, q-PCR validated the expression of part of SMAD family targets, which were identified to express differentially in response to PDGF-BB *via* RNA-sequencing; BMPR2 was detected in RAPSMCs treated with PDGF-BB (time course and dose course) *via* Western blot at protein level **c** and qRT-PCR at mRNA level **d**, **e**, *n* = 3, *p < 0.05, **p < 0.01 *vs* 0 h or 0 ng/ml

Our interest focused on the proliferation, migration and differentiation of PASMCs, hence those differentially expressed targets of SMADs involved in these processes were chosen for validation *via* qRT-PCR (Fig. 4a). The results showed that, *bambi, edn1* and *id2* presented time-dependent downregulation in response to PDGF-BB treatment. On the other hand, *myc, cdkn1a, jun, col2, tgfb1, gja1, col5a1* and *ctnnb1* displayed time-dependent upregulation in response to PDGF-BB treatment. The results from qRT-PCR assays were consistent with RNA-sequencing data, except for *myc* (Fig. 4b).

Taken all results above together, we revealed a significant role of SMAD1 in regulating PDGF-BB induced gene expression. Next we would try to explore how PDGF-BB impacts SMAD1 to regulate the corresponding gene expression.

### Downregulation of BMPR2 led to expression alteration of SMADs targets

To explore what accounted for SMAD1-involved gene expression in PDGF-BB stimulated RPASMCs, data from RNA-sequencing and iTRAQ assay were analyzed and BMPR2 was found to be downregulated in response to PDGF-BB treatment both in mRNA and protein levels. As well known, BMPR2 is the core upstream regulator in BMPR2/SMADs signaling. Through BMRP2-dependent BMRP1 activation, SMAD1 translocates into nucleus and interacts with other transcription factors to regulate gene expression, negatively or positively [28]. Western blot confirmed that BMPR2 protein downregulated in response to PDGF-BB with time-dependent manner in RPASMCs (Fig. 4c). qRT-PCR also validated that BMPR2 mRNA altered with time- and dose- dependent manner in response to PDGF-BB in RPASMCs (Fig. 4d, e). Taken together, theses data suggested that PDGF-BB induced BMPR2 downregulation could be causative of expression alteration of SMADs targets.

### miR-376b mediated the downregulation of BMPR2

**Fig. 5:**
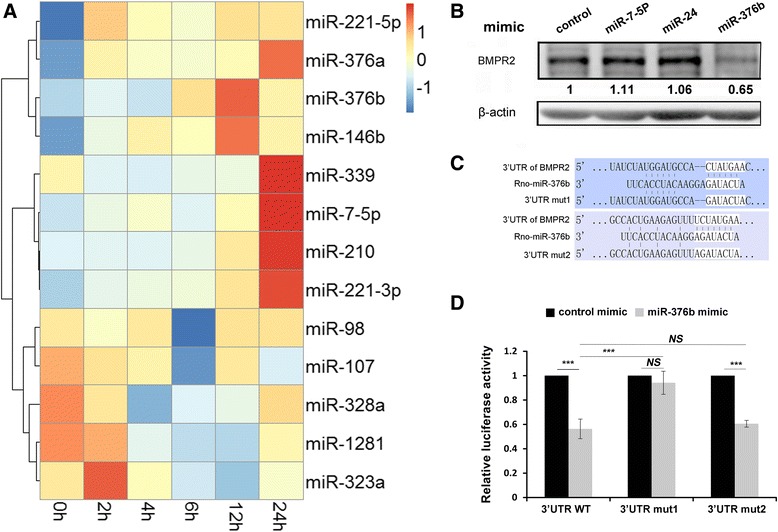
BMPR2 is a potential direct target of miR-376b. **a**, Heatmap showed relative level of miRNAs following PDGF-BB treatment for different time; **b**, BMPR2 protein level was detected in RPASMCs transfected with miRNA mimics *via* Western Blot; **c**, The two conserved miR-376b binding sites in the 3′-UTR of BMPR2 along with the mutation sites, respectively; **d**, 3′-UTR luciferase reporter assay with target sites and their mutant along with miR-376b/miR-Con mimics. Bar charts of luciferase reporter analysis represent means ± SD (*n* = 3), *p < 0.05, **p < 0.01 *vs* miR-Con/3′UTR of targets

To determine if PDGF-BB regulates BMPR2 expression in post-transcriptional level, we next explored whether miRNAs involved in downregulation of BMPR2. Our lab has investigated the expression profiling of 1078 miRNAs and identified a group of miRNAs differentially expressing in HPASMCs treated by PDGF-BB [28]. Among them, 13 miRNAs were significantly changed in both human and rat PASMCs, with 7 (miR-221-5p, miR-221-3p, miR-376a, miR-376b, miR-146b, miR-7-5p, miR-210) upregulated and 6 (miR-339, miR-98, miR-107, miR-328a, miR-1281, miR-323a) downregulated in response to PDGF-BB (Additional file 5). Further study suggested that expression of these 13 miRNAs also changed with time-dependent manner following PDGF-BB treatment in RPASMCs (Fig. 5a).

Using online prediction tool of Targetscan (http://www.targetscan.org/), multiple binding sites of miR-7, miR-24 and miR-376b respectively were identified in BMPR2 3’UTR. To test whether BMPR2 is a direct target of these 3 miRNAs, the effect of miRNA mimic on BMPR2 protein level was detected firstly. Western blot showed that only miR-376b mimic could inhibit BMPR2 protein level (Fig. 5b). In addition, HEK-293 cells that co-transfected with miR-376b mimic and a luciferase construct containing sequence of BMPR2 3’UTR resulted in significant decrease in luciferase activity (Fig. 5d), but not miR-7 and miR-24 (data not showed). Two potential miR-376b binding sites within BMPR2 3’UTR have been identified *via* Targetscan (Fig. 5c), however, they exhibited great difference in their susceptibility to miR-376b mediated repression. Mutation on the first binding site (Mut1) completely abolished the inhibitory effect caused by miR-376b, but not the second binding site mutated (Mut2) (Fig. 5d). Together, these data demonstrated that upregulation of miR-376b in response to PDGF-BB repressed the BMPR2 protein level post-transcriptionally.

### miR-376b promoted proliferation of RPASMCs

**Fig. 6:**
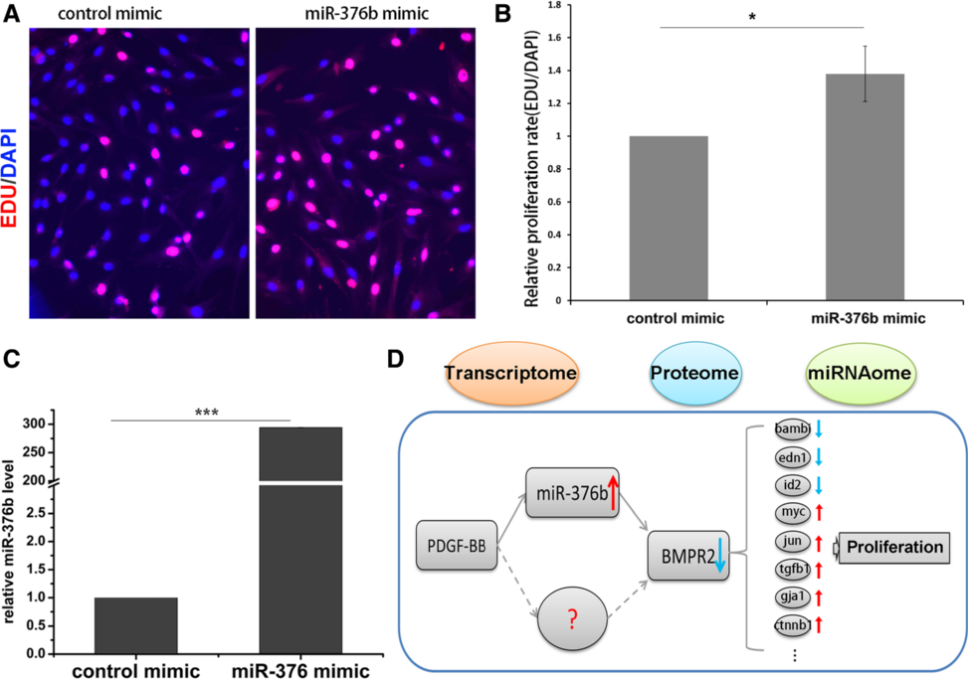
miR-376b promoted RPASMCs proliferation. **a**, RPASMCs transfected with miR-376b or control mimic were incubated with EDU solution for 4 h, and then were fixed and stained with Apollo dye (*red*) and DAPI (*blue*); **b**, Bar charts showing relative EdU incorporation rate in miR-376b transfected RPASMCs, *n* = 3, data are shown as mean ± SD. *p < 0.05 *vs* control mimic; **c**, Bar charts showing quantification of miR-376b in transfected RPASMCs. *n* = 3, data are shown as means ± SD, ***p < 0.001 *vs* control; **d**, Model depicting PDGF-BB promoted proliferation *via* regulating miR-376b-induced BMPR2 decrease

A series of reports have suggested that downregulation of BMPR2 promoted proliferation of PASMCs [4]. Therefore, we investigated whether miR-376b could impact proliferation of RPASMCs. EdU incorporation assay showed that, miR-376b mimic significantly promoted proliferation of RPASMCs (Fig. 6a, b). This data further demonstrated that miR-376b could inhibit BMPR2 expression and promote proliferation of RPASMCs.

In summary, this study integrated transcriptome and proteome and discovered that, in response to PDGF-BB, miR-376b was upregulated to repress BMPR2 level. Decreased BMPR2 further impact downstream SMADs and altered target genes expression, which promoted proliferation of RPASMCs (Fig. 6d).

## Discussion

PDGF signaling plays important role in pulmonary vascular remodeling, however, the detail mechanism underlying this process remains elusive. There has been a set of genome-wide studies about PDGF-responsive gene expression in VSMCs, which mainly based on traditional high throughput technology, such as microarray, covering only portion of the whole transcriptome [21, 22]. Moreover, the four fundamental cellular processes, including transcription, mRNA degradation, translation and protein degradation, make the correlation between mRNA and protein more complicated. Therefore, integrated analysis of mRNA and protein levels would provide more comprehensive information to understand gene regulation [29]. However, integrating transcriptome and proteome in VSMCs is still lacking [23]. In this study, we utilized the next generation sequencing, combining iTRAQ isobaric labeling technology to globally study the gene expression regulation in PASMCs in response to PDGF-BB. Our results confirmed the crucial role of transcription factors JUN and MYC in PASMCs proliferation and migration, as previously suggested [23]. Moreover, our integrated analysis extended the understanding of PDGF-BB function on PASMCs, revealing the crosstalk between PDGF signaling and BMPR2/SMADs axis mediated by miR-376b. Previous report suggested that BMP/BMPR2/SMADs signaling prevented PDGF-BB–induced proliferation of PASMCs by decreasing nuclear phospho-ERK *via* its transcriptional target apoE [30]. Our work suggested that PDGF signaling could regulate BMPR2/SMADs signaling and hence promote the proliferation of PASMC in the other hand.

Integrated analysis indicated that PDGF-BB impacted targets expression of 10 transcription factors, namely HIF1A, JUN, EST1, ETS1, SMAD1, FOS, SP1, STAT1, LEF1 and CEBPB (Fig. 3c), both in mRNA and protein level. Mutations or downregulation of BMPR2 is presented in most cases of heritable PAH (HPAH), indicating a functional role of BMPR2 in PAH initiation/progression [4]. Hence we focused on exploring the effect of PDGF-BB on BMP/BMPR2/SMADs signaling. In this study, we identified 11 alternatively expressed genes that are potential targets of BMP/BMPR2/SMADs axis. Among them *bambi, edn1* and *id2* were downregulated in a time-dependent manner, while *myc, cdkn1a, jun, col2, tgfb1, gja1, col5a1* and *ctnnb1* were upregulated (Fig. 4b). These genes have been annotated with important roles in mediating the proliferation, migration and differentiation of PASMCs [23, 31–38], and our finding further suggested their cooperation in mediating the PDGF-induced dysfunction of PASMCs. For instance, since *bambi* negatively regulates TGF-β-family signaling [31], *bambi* downregulation and *tgfb1* upregulation indicated that PDGF signaling could positively regulate TGF-β signaling. Previous reports showed that TGF-β signaling inhibits PASMCs proliferation in normal condition but promotes proliferation in BMPR2-mutated PASMCs [39]. Therefore, activated PDGF signaling in PASMCs would lead to increased TGF-β signaling and reduced BMP signaling, both of which are implicated in PAH pathogenesis. HIF1A is another TF deserved paying attention to, since many reports have suggested the important role of HIF1A in pulmonary vascular remodeling [26, 27]. Our analysis revealed that, differentially expressed genes in response to PDGF-BB were enriched the most significantly in HIF1A, in both mRNA and protein levels (Fig. 3c). Hence, our data indicated the tight connection between PDGF signaling and hypoxia, which has received little attention [40].

Few researches have focused on the regulation of BMPR2 expression in PASMCs. A report suggested that miR-21 potentially suppressed BMPR2 protein level [41]. MicroRNA (miRNA) is a group of small endogenous noncoding single-strand RNAs (~21–25 nt), which negatively regulate gene expression in translation phase [42]. miRNA has been emerged as key player in cardiovascular diseases and cancer development and progression and, more recently, in PAH pathogenesis [42]. Several groups has globally explored the differentially expressed miRNAs in response to PDGF-BB in PASMCs. For example, Brandi N. D and his colleagues cloned and sequenced miRNAs expressed in PASMCs under vehicle- or PDGF-BB-treated conditions and found that miR-221 was one of the few miRNAs enriched in PDGF-BB-treated PASMCs, which served as a modulator of the phenotypic change of PASMCs *via* targeting c-Kit and p27Kip1 [43]. Li et al. colleagues performed miRNA microarray analysis in human aortic smooth muscle cells (SMCs) stimulated with PDGF-BB and identified miR-638 as one of the most significantly downregulated miRNA in human VSMCs in response to PDGF-BB stimulation [7]. Together, these data highlighted the contribution of the miRNA regulation in PDGF signaling pathway. Our lab utilized an improved miRNA detection assay, S-Poly (T) plus assay, to profile the expression of 987 miRNAs and found 13 miRNAs with altered expression in HPASMCs after PDGF-BB stimulation. To investigate whether miRNAs involved in PDGF-BB induced BMPR2 downregulation, online software Targetscan were used to predict the binding sites of those 13 miRNAs, 3 of which possessed the potential binding sites in the 3’UTR of BMPR2. However, Western blot showed that only miR-376b mimic could inhibit the expression of BMPR2 protein.

Our data suggested that PDGF-BB treatment induced downregulation of BMPR2 not only at protein level but also at mRNA level. miR-376b could inhibit the expression of BMPR2 at protein level (Fig. 5b), however, BMPR2 mRNA in PASMCs remained unchanged after miR-376b mimic transfection (data not shown), suggesting the inhibitory effect of miR-376b on BMPR2 is posttranscriptional. As to the transcriptional regulation of BMPR2 expression, previous study showed that BMPR2 transcription could be affected by methylation and acetylation in endothelial cells [44]. However, it appeared not the case in PASMC upon PDGF stimulation, as suppressed methylation with 5-Azacytidine and deacetylation with Trichostatin A failed to recover the BMPR2 mRNA levels inhibited by PDGF-BB (data not shown). Therefore, we hypothesize that PDGF-BB regulates BMPR2 transcription through impacting promoter activity and further investigation is needed to verify this hypothesis.

## Conclusion

In conclusion, our results provided the first systems-level integrated analysis of PDGF-BB-regulated transcriptome, proteome and miRNAome (3-omics) in PASMCs. The results demonstrated that PDGF-BB-induced miR-376b upregulation mediated BMPR2 downregulation, which led to expression change of its target genes and promoted proliferation of PASMCs.

## Method

### Ethics statement

All experiments were carried out according to China Council on Animal Care and the protocols used were approved by the Animal Care and Use Committee of Guangdong Province, China.

### Cell culture and transfection

RPASMCs were generous gifts from Dr. Zeng Yan, and cultured as previously reported [45]. Cultured primary RPASMCs (≤4 passage) were starved for 12 h (h) and then treated with PDGF-BB (30 ng/ml, R&D system, Minneapolis, MN) for different time (0, 2, 4, 6, 12, 24 h). miRNA mimics (20nM) were transfected with K2 transfection reagent (Biontex, Planegg, Germany) in RPASMCs. HEK-293a, a cell line derived from human embryonic kidney, was purchased from American Type Culture Collection (Manassas, USA). The transfection of miRNA mimic and DNA plasmids into HEK-293 cells were performed with polyethylenimine (PEI, Geneups, Shenzhen, China).

### RNA deep-sequencing and identification of differentially expressed mRNA

Total RNAs were extracted with RNAiso Plus (Takara biotechnology Co., Dalian, China). Following procedure was performed by the sequencing company (Novogene Bioinformatics Technology Co., Beijing, China). Briefly, Sequencing libraries were generated using the rRNA-depleted RNAbyNEBNext® Ultra™ Directional RNA Library Prep Kit for Illumina® (NEB, USA) following manufacturer’s recommendations. The libraries were sequenced on an Illumina Hiseq 2000 platform and 100 bp paired-end reads were generated. Demultiplexed and quality filtered reads were then aligned to Rat reference assembly (Rn5) using TopHat (v.2.0.9) [46]. The mapped reads of each sample were assembled by both Scripture (beta2) [47] and Cufflinks (v2.1.1) [48] in a reference-based approach.

Cuffdiff (v2.1.1) was used to calculate FPKMs (fragments per kilo-base of exon per million fragments mapped) of coding genes in each sample [48]. Gene FPKMs were computed by summing the FPKMs of transcripts in each gene group. Cuffdiff provides statistical routines for determining differential expression in digital transcript or gene expression data using a model based on the negative binomial distribution [48]. Genes with a Q value < 0.05, FKPRM > 0.5 and FC > 2 were assigned as differentially expressed.

### GO and KEGG analysis

GO-term analysis was utilized to determine the potential functions of differentially expressed genes in response to PDGF-BB. GO term with Q-values ≤ 0.05 was considered to be significantly enriched. Meanwhile, KEGG analysis was used to assess the pathway that differential genes may involve in. KEGG term with Q-values ≤ 0.05 was considered to be significantly enriched. Both GO and KEGG analysis were performed with KOBAS 2.0 online software (http://kobas.cbi.pku.edu.cn/) [24].

### Identification of key transcription factors (TFs) regulating differentially expressed genes

To identify key TFs, TF-target interaction data for 179 TFs were collected from public database TRED to build a expanded list of rat TF-target interaction, which includes homologous human and mouse ones [25]. We utilized the enrichment level to valuate the significance of TFs in PDGF-BB regulating gene expression, which was assessed with *Fisher’s exact test*, and the FDR correction method is “*Benjamini and Hochberg*”.

### iTRAQ labeling and Identification of differentially expressed proteins

ITRAQ labeling was performed using the 8-plex iTRAQ reagents (Applied Biosystems, Foster City, CA, USA) as previously reported [49]. Then, the labeled samples were lyophilized and assigned to HPLC-MS/MS (high performance liquid chromatography-mass spectrometry/ mass spectrometry) analysis in HPLC electrospray ionization MS/MS system (Applied Biosystems, Foster City, CA, USA).

Quality assessment of the iTRAQ datasets was performed as described [49]. The statistical differences between two groups were assessed with the double-sided Student’s *t* test. Additionally, fold-change = average (12 or 24 h) / average (0 h). The differentially expressed proteins were identified using the following criteria: 1) overall *P* values are less than 0.05; 2) proteins quantified in at least two replicates; and 3) absolute fold changes are larger than 1.6.

### Quantitative RT-PCR

For mRNA detection, total RNA was reversely transcribed using M-MLV Reverse Transcriptase (TaKaRa, Dalian, China) with oligo(dT)_18_ plus random hexamer primers (Promega, Madison, WI). qRT-PCR was performed with gene specific primers and SYBR Green PCR Master Mix (Applied Biosystems, Foster City, CA) on ABI StepOne real-time PCR System (Applied Biosystems, Foster City, CA, USA). The expression level of each gene was normalized to internal control β-actin gene and the expression level of each mRNA was calculated using the 2^(−ΔΔCT)^ method. The S-Poly(T) Plus method was used for miRNA detection as previously reported [50]. The snoRNA-202 was used as an internal control. Primers used for reverse transcription and qRT-PCR were summarized in Additional file 6.

### Western blot

Cells were lysed with ice-cold RIPA (50 mM Tris–HCl, pH 7.5; 150 mM NaCl; 1 % NP-40; 0.25%sodium deoxycholate, 1 mM EDTA) buffer supplemented with protease inhibitor cocktail (Sigma-Aldrich Inc., St. Louis, MO). Each sample with 30 μg protein were then electrophoresed on the sodium dodecyl sulfate (SDS) polyacrylamide gel and then electroblotted to nitrocellulose filter membranes (Millipore, Bedford, MA). Membranes were immersed in blocking buffer (5 % degreased milk powder) for 1 h and incubated with antibodies against BMPR2 (ProteinTech Group, Chicago, IL), β-actin (SantaCruz Biotechnology, Santa Cruz, CA) overnight at 4 °C. Next, the membranes were washed and incubated with horseradish peroxidase-conjugated secondary antibodies (Jackson Immuno-Research, West Grove, PA) for 1 h at room temperature. The protein bands were visualized with the SuperSignal chemiluminescent detection module (Pierce, Thermo Scientific Inc., San Jose, CA).

### 3′UTR luciferase reporter assay

TargetScan algorithm (http://www.targetscan.org) was applied to predict targets and the miRNA binding sites. The 3′UTR of BMPR2 were PCR amplified and inserted into pGL4-plasmid (Promega). The corresponding mutant constructs with six mutated residues in the region of seeding sequence were generated by site-directed mutagenesis. Luciferase activity was measured in cell extracts with a Lumat LB9508 luminometer (Berthold, Bad Wildbad, Germany). The primers used were as follow: cloning 3′ UTR of BMPR2: 5- GGA ATT CCC CGC CTT GTT ATC AGT CG-3 (forward) and 5- CCG CTC GAG TTA CAG CAA GCC TTT TAA ACC T −3 (reverse); BMPR2-mut1, 5-GAT GCC AGA TAC TAC GCT GAC ATT AAG CCA CTG A-3 (forward) and 5-TCA GCG TAG TAT CTG GCA TCC ATA GAT AAT ACA AAA G-3 (reverse); BMPR2-mut2, 5- GAA GAG TTT AGA TAC TAT AAG TGT AAG TAA ATG CTT TG-3 (forward) and 5- TAC ACT TAT AGT ATC TAA ACT CTT CAG TGG CTT AAT G −3 (reverse).

### Proliferation measurement (EdU incorporation assay)

EdU labeling was performed using the EdU Assay Kit (Ribobio, Guangzhou, China) as recommended by the manufacturer. Briefly, approximately 1 × 10^4^ cells were seeded intriplicate in 48-well plates, and the cells were cultured for 24 h and then transfected with miR-376b or control mimic for 48 h, then exposed to 20 μM EdU for 4 h at 37 °C. The cells were then fixed in 4 % paraformaldehyde for 30 min at room temperature and permeabilized in 0.5 % Triton X-100 for 10 min. Cells were washed with PBS, and each well was incubated with 200 μl 1 × Apollo^®^ reaction cocktail for 30 min. DNA was then stained with 1 μg/ml DAPI (200 μl per well) for 30 min and imaged under a fluorescent microscope. All data are shown as a percentage of control mimic.

### Statistical analysis

Except for those omics data, all data shown are mean values of at least three experiments with standard deviation (SD). Correlation test was performed with Pearson method. When only two groups were compared, the statistical differences were assessed with the double-sided Student’s *t* test. Significant differences between groups were analyzed using one-way ANOVA. A *p* value less than 0.05 was considered significant.

## Additional files

Additional file 1: Table S1. Differentially expressed mRNA in RPASMCs treated with PDGF-BB for 12 h. (XLSX 185 kb)
Additional file 2: GO and KEGG analysis for differentially expressed mRNA. (XLSX 956 kb)
Additional file 3: Table S2. Differentially expressed protein in RPASMCs treated with PDGF-BB for 12 h. (XLSX 33 kb)
Additional file 4: Table S3. Table S2. Differentially expressed protein in RPASMCs treated with PDGF-BB for 24 h. (XLSX 42 kb)
Additional file 5: Differentially expressed miRNA in HPASMCs. (XLSX 9 kb)
Additional file 6: Primers for qRT-PCR. (XLSX 15 kb)

## Abbreviations

FC: Fold change
FDR: False discovery rate
FPKMs: Fragments per kilo-base of exon per million fragments mapped
GO: Gene ontology
HPAH: Heritable PAH
HPASMCs: Human PASMCs
HPLC-MS/MS: High performance liquid chromatography-mass spectrometry/ mass spectrometry
iTRAQ: Relative and absolute quantification
KEGG: Kyoto Encyclopedia of Genes and Genomes
MCT: Monocrotaline
PAH: Pulmonary arterial hypertension
PASMCs: Pulmonary arterial smooth muscle cells
PDGF-BB: Platelet-derived growth factor BB
PDGFR: PDGF receptor
qRT-PCR: Quantificational Real-time Polymerase Chain Reaction
RPASMCs: Rat PASMCs
SD: Standard deviation
TF: Transcription factor
UTR: Untranslated regions
VSMCs: Vascular smooth muscle cells.
